# Developmental Expression of Membrane Pumps and Ion Channels in Human Vestibular Endolymph Homeostasis

**DOI:** 10.1002/dneu.70010

**Published:** 2026-01-25

**Authors:** Edward S. A. van Beelen, Wouter H. van der Valk, John C. M. J. de Groot, Peter Paul G. van Benthem, Heiko Locher

**Affiliations:** ^1^ OtoBiology Leiden, Department of Otorhinolaryngology and Head & Neck Surgery Leiden University Medical Center Leiden the Netherlands; ^2^ The Novo Nordisk Foundation Center for Stem Cell Medicine (reNEW) Leiden University Medical Center Leiden the Netherlands

**Keywords:** dark cells, endolymph, fetal development, human vestibular system, inner ear, ion channels, membrane pumps

## Abstract

The expression patterns of key membrane pumps and ion channels involved in endolymph cycling have been studied in the rodent inner ear and the developing and adult human cochlea. However, little is known about their expression during the development of the human vestibular system. In this study, we provide a comprehensive overview of expression profiles of ion pumps, cotransporters, and exchangers in the developing human utricle and ampullae from fetal week (FW) 8 to 17. Immunohistochemistry analysis revealed that ATP1A1 and ATP1B2 co‐localize at the basolateral membranes of dark cells. In addition, BSND expression was observed in transitional cells and dark cells in both the ampulla and utricle from FW10. We further characterized the expression of gap junction proteins (GJA1, GJB2, and GJB6) and found that KCNQ1 was expressed by transitional cells and dark cells starting from FW14. SLC12A2 immunostaining was detected in dark cells around FW10. Lastly, we investigated the spatiotemporal expression of pendrin. These detailed observations of protein expression during human inner ear development enhance our understanding of endolymph homeostasis.

## 1 Introduction

**FIGURE 1:**
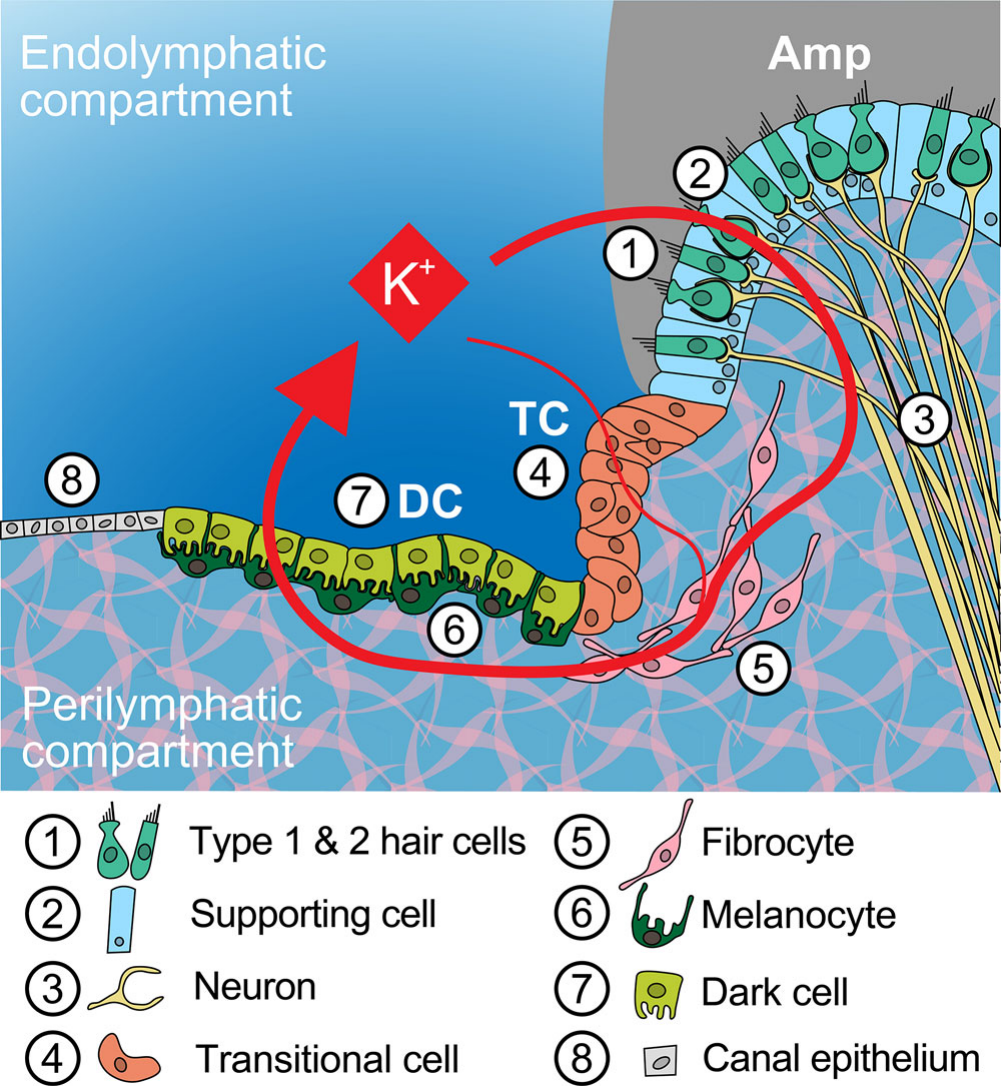
**Schematic detail of the cell types involved in potassium recycling in the human ampulla**. Potassium recycling in the adult vestibular organs occurs through a sophisticated set of ion transporters, including ion pumps (active transport), ion channels (passive transport), and gap junctions (simple and facilitated diffusion). Stereocilia in the apical membranes of the hair cells (#1) contain mechanoelectrical transduction channels that open upon deflection of the stereociliary bundles, thereby allowing influx of K^+^ and subsequent depolarization of the hair cell. Voltage‐gated Ca^2+^ channels in the basolateral membranes open and the subsequent influx of Ca^2+^ causes release of neurotransmitter molecules into the synaptic cleft, resulting in excitation of the afferent neurons (#3). The membrane potential returns to its resting phase by the release of K^+^ into the intercellular space through voltage‐gated K^+^ channels (#5), from which it diffuses to neighboring cells: The subepithelial melanocytes (#6), transitional cells (#4) and dark cells (#7). Transitional cells and dark cells take up K^+^ by active transport at their basolateral membranes via Na^+^/K^+^‐ATPases and Na^+^‐K^+^‐2Cl^−^ cotransporters. Ultimately, K^+^ is recycled to the endolymph by means of K^+^ channels, thereby concluding the cycle (Wangemann 2002).

Electrolyte homeostasis of vestibular endolymph and perilymph is crucial for the proper functioning of the vestibular organs, that is, the saccule, utricle, and the ampullae of the semicircular canals. These fluids are separated by specialized epithelia containing sensory cells (hair cells) and various non‐sensory cells involved in electrolyte regulation (Figure 1). Non‐sensory cells surround the sensory epithelium and can be categorized into a transitional zone containing transitional cells, and a dark cell area containing dark cells (absent in the saccule) and subepithelial melanocytes. In early developmental stages, a prosensory domain is defined, fated to generate sensory hair cells and associated supporting cells. Together, these epithelia delineate a loosely connected mesenchyme, through which perilymph flows (Köppl et al. 2018). Each cell type exhibits a unique expression pattern of ion pumps, ion channels, and gap junctions involved in electrolyte cycling.

The significance of electrolyte regulation in endolymph homeostasis is underscored by mutations in genes coding for proteins involved in the recycling process, resulting in sensorineural hearing loss (SNHL) and/or balance disorders (Table 1). Genes include ATP1A1, ATP1B2, GJB2, GJB6, GJA1, KCNQ1, SLC26A4, SLC12A2, and BSND.

**TABLE 1:** Selected genes and their related syndromes/inner ear symptoms.

Gene	Protein	Related syndrome / symptoms	Involved ions
ATP1A1	Na^+^/K^+^‐ATPase α1 subunit		3 sodium ions out, 2 potassium ions in
ATP1B2	Na^+^/K^+^‐ATPase β2 subunit		3 sodium ions out, 2 potassium ions in
GJB2	Connexin 26	DFNB1A / DFNA3 / DFNB1	Passage of K+, Na+, Ca2+, Cl‐
GJB6	Connexin 30	DFNA3	Passage of K+, Na+, Ca2+, Cl‐
GJA1	Connexin 43	Hearing loss	Passage of K+, Na+, Ca2+, Cl‐ (assumed)
KCNQ1	KVLQT1	Jervell and Lange‐Nielsen syndrome	K+ ions out
SLC26A4	Pendrin, Cl‐/HCO3‐ exchanger	DFNB4 / Pendred syndrome	Anion transport, HCO3‐ ions out, Cl‐ ions in
SLC12A2	Na^+^/K^+^/2Cl^−^ cotransporter, NKCC1	Hearing loss, balance disorders	Na+, K+, 2Cl‐ ions in
BSND	Barttin	Bartter syndrome type IV	Cl‐ ions out

While the distribution of key ion pumps and channels involved in ion transport has been extensively studied in the mouse cochlea (Wangemann and Marcus 2017) and during fetal development of the human cochlea (Locher et al. 2015), the expression patterns in the developing human vestibular system remain, to the best of our knowledge, unexplored. Addressing this gap has implications for advancing translational research since mutations in genes associated with deafness often also lead to vestibular dysfunction, reflecting shared protein expression patterns between the auditory and vestibular system (Mei et al. 2021). We examine the expression of key ion pumps, cotransporters, and exchangers involved in endolymph production in the human vestibular system from FW8 to FW17.

## 2 Material and Methods

### 2.1 Ethics Statement

Use of human embryonic and fetal specimens was in accordance with Dutch legislation and the WMA Declaration of Helsinki guidelines, and approval for this project was obtained from the Medical Research Ethics Committee of Leiden University Medical Center (protocol registration number B18.044). Written informed consent of the donors was obtained following the Guidelines on the Provision of Fetal Tissue set by the Dutch Ministry of Health, Welfare and Sport (revised version, 2018). For privacy and ethical reasons, the motivation for termination was not recorded in the dataset.

### 2.2 Specimen Collection and Processing

Intact human embryonic and fetal inner ears were collected after elective termination of pregnancy by vacuum aspiration. Embryonic or fetal age (in weeks, FW), defined as the duration since fertilization, was determined using obstetric ultrasonography based on the Crown‐Rump Length measurement prior to termination, with two weeks subtracted to estimate fetal age. This method has a standard error of ±2 days. Specimens with known or visible developmental abnormalities were excluded. Tissue was obtained at the following developmental stages: FW8 (*n* = 2), FW10 (*n* = 3), FW12 (*n* = 4), FW14 (*n* = 2), FW16 (*n* = 1), and FW17 (*n* = 1). Time between termination and collection was kept to a minimum of several minutes. Inner ears were processed as previously described (Locher et al. 2013). Briefly, inner ears were harvested from vacuum‐aspirated tissue, collected in phosphate‐buffered saline pH 7.4 (PBS), transferred to 4% formaldehyde (prepared from paraformaldehyde) in 0.1 M Na^+^/K^+^‐phosphate buffer (pH 7.4) and fixed for at least one night at 4°C. Inner ears from FW12 and older were decalcified for 1–3 weeks in 10% EDTA.2Na (Sigma‐Aldrich, St. Louis, MO, USA) in distilled water (pH 7.4) at 4°C. All specimens were subsequently dehydrated in an ascending ethanol (70%–99%) series, cleared in xylene and embedded in paraffin wax.

### 2.3 Histology and Immunohistochemistry

Sections (5 µm) were cut using an HM 355 S rotary microtome (Thermo Fisher Diagnostics B.V., Landsmeer, the Netherlands) and transferred to aminosilane‐coated glass slides and air‐dried overnight at room temperature. Sections were deparaffinized in xylene and rehydrated in a descending series of ethanol (96%–50%) and several rinses in deionized water. Every 10–20 sections, one section was selected for routine staining with hematoxylin and eosin (H&E).

Antigen unmasking was performed in 10 mM sodium citrate buffer (pH 6.0) for 12 min in a microwave oven set at 97°C. After rinsing in washing buffer (0.05% Tween‐20 [Promega, Madison, WI, USA] in PBS), sections were incubated for 30 min with blocking solution (5% bovine serum albumin [BSA; Sigma‐Aldrich, St. Louis, MO, USA] and 0.05% Tween‐20 in PBS) followed by overnight incubation at 4°C with the following primary antibodies, appropriately diluted in blocking solution, either as single, double or triple immunostainings: mouse anti‐ATP1A1 (1:200, Novus Cat# NB300‐146, RRID:AB_2060979), rabbit anti‐ATP1B2 (1:100, Novus Cat# NBP2‐97186), rabbit anti‐BSND (1:1000, Novus Cat# NBP2‐49101), mouse anti‐GJA1 (CXN‐6, 1:100, Thermo Fisher Scientific Cat# MA1‐25097, RRID:AB_779902), rabbit anti‐GJB2 (1:100, Alomone Labs Cat# ACC‐212, RRID:AB_11124274), rabbit anti‐GJB6 (1:100, Thermo Fisher Scientific Cat# PA5‐11640, RRID:AB_2111053), rabbit anti‐KCNQ1 (1:20, Atlas Antibodies Cat# HPA048553, RRID:AB_2680438), chicken anti‐SLC12A2 (1:200, Novus Cat# NB100‐75623, RRID:AB_1049234), and rabbit anti‐SLC26A4 (1:100, Novus Cat# NBP1‐85237, RRID:AB_11032075) immunoglobulins. Next, the sections were incubated for 2 h at room temperature with the following Alexa Fluor‐conjugated secondary antibodies: AF488 donkey anti‐rabbit (Abcam Cat# ab150061, RRID:AB_2571722), AF488 donkey anti‐goat (Abcam Cat# ab150133, RRID:AB_2832252), AF594 donkey anti‐mouse (Thermo Fisher Scientific Cat# A‐21203, RRID:AB_2535789) and AF680 donkey anti‐rabbit (Thermo Fisher Scientific Cat# A10043, RRID:AB_2534018) immunoglobulins, diluted at 1:500 in blocking solution. Nuclei were stained with 4′,6‐diamidino‐2‐phenylindole (DAPI; 1:1000, Vector Laboratories Ltd., Peterborough, UK). Sections were mounted with ProLong Gold Antifade Mountant (Thermo Fisher Scientific, Waltham, MA, USA). Negative controls were carried out by matching isotype controls and also by omitting primary antibodies. Positive controls were carried out by staining sections of known positive human tissue samples. At least three separate immunostainings were performed with each primary antibody.

### 2.4 Image Acquisition and Processing

Sections stained with H&E were digitized using a Pannoramic MIDI scanner and viewed with CaseViewer software (3DHISTECH, Budapest, Hungary). Pseudocolor images of immunostained sections were acquired with a Leica SP8 confocal laser scanning microscope using Leica objectives (20x/0.7 dry HC PL Apo, 40x/1.3 oil HC PL Apo CS2, 63x/1.4 oil HC PL Apo and 100x/1.3 oil HC PL Fluotar), operating under Leica Application Suite X microscope software (LAS X, Leica Microsystems, Buffalo Grove, IL, USA). Maximal projections were obtained from image stacks with optimized z‐step size. Brightness and contrast adjustments were performed with Fiji (ImageJ version 1.52p) or Adobe Photoshop CC 2018. Colors were adjusted for colorblindness. If not shown, separate channels of merged immunostaining images can be provided by the corresponding author upon request.

## 3 Results

Expression patterns of investigated proteins were identical between utricle and ampulla, unless otherwise stated. Figures show expression in either utricle or ampulla based on most representative results.

### 3.1 Membrane Pump Subunits ATP1A1 and ATP1B2 Co‐Localize at the Basolateral Membranes of Dark Cells in the Utricle and Ampulla

**FIGURE 2:**
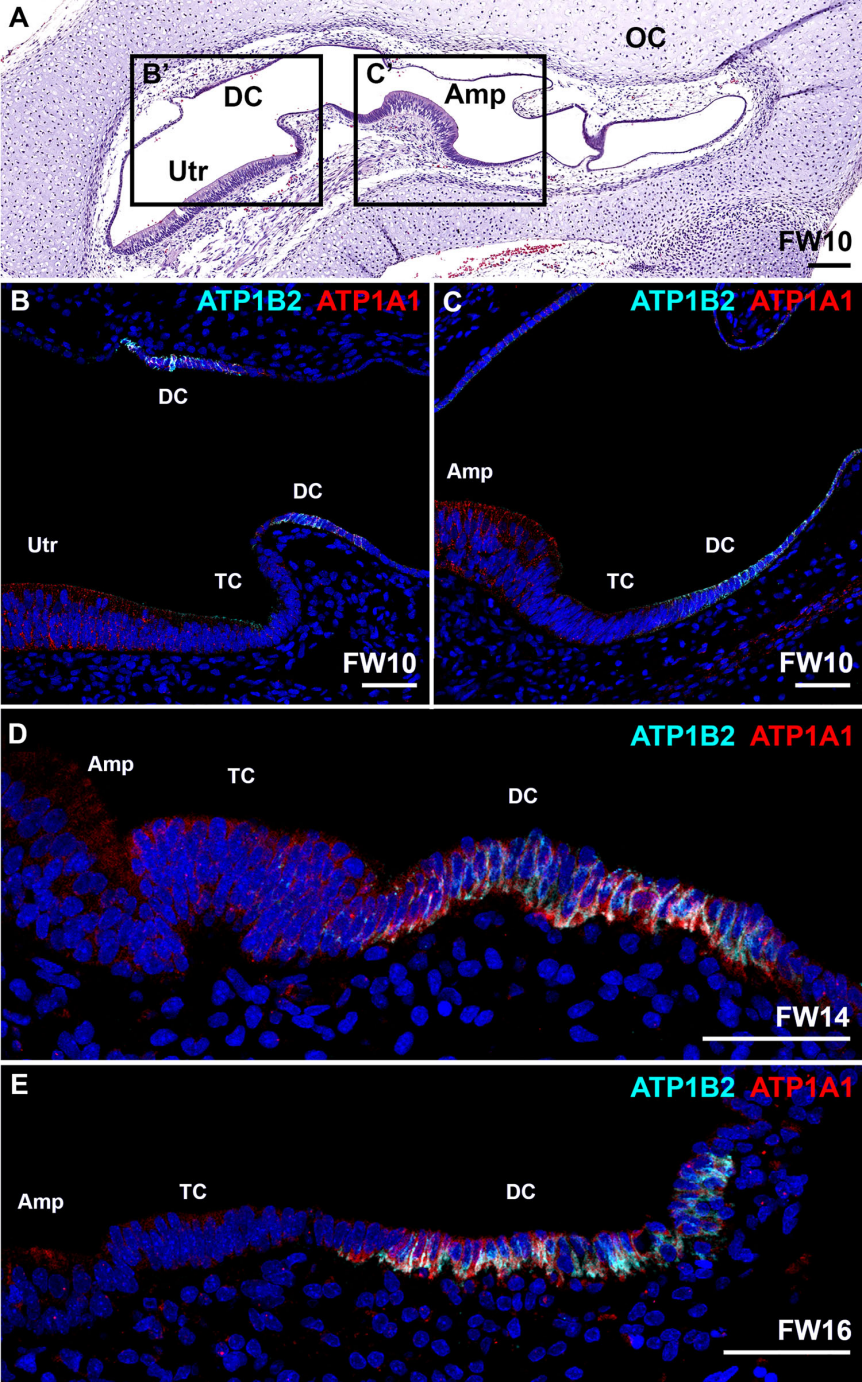
Expression of ATP1A1 and ATP1B2 in the developing utricle and ampulla. (A) H&E overview of the developing vestibular system at FW10. (B–C) At FW10, immunostaining for ATP1A1 was observed in the sensory domain, non‐sensory epithelia and the subepithelial mesenchyme of the utricle and the ampulla. Expression of ATP1B2 was seen in the dark cells and transitional cells of the utricle and the ampulla. B–C correspond to areas B’‐C’ in the H&E overview. (D) At FW14, expression of ATP1A1 in the ampulla was predominantly seen in the basolateral membranes of the dark cells. Some immunostaining was visible in the transitional cells, whereas immunostaining of ATP1B2 was confined to the basolateral membranes of the dark cells. (E) Immunostaining of ATP1A1 and ATP1B2 co‐localize at the basolateral membranes of the dark cells. Amp, sensory domain of the ampulla; DC, dark cells; OC, otic capsule; TC, transitional cells; Utr, sensory domain of the utricle. Cyan: ATP1B2; red: ATP1A1. Scale bars: 200 µm (A), 50 µm (B–E).

At FW10, immunostaining for ATP1A1 was seen in the sensory domains, the dark cell epithelia and, to a lesser degree, in the transitional cells and subepithelial mesenchyme of the utricle and ampulla (Figure 2A–C; Figure S1A,C). At this stage, expression of ATP1B2 was most evident in dark cells, but transitional cells showed some expression as well (Figure 2B,C; Figure S1B,D). From FW14‐16, expression of ATP1A1 was predominantly seen at the basolateral membranes of the dark cells, whereas expression in the sensory domain of the ampulla and the transitional zone gradually diminished (Figure 2D–E; Figure S1E,G). At this stage, ATP1B2 was confined to the basolateral membranes of the dark cells (Figure 2D–E; Figure S1F,H). ATP1A1 and ATP1B2 co‐localize at the basolateral membranes of the dark cells in the utricle and ampulla.

### 3.2 Transitional Cells and Dark Cells in the Ampulla Express Barttin Protein BSND From FW10 Onwards

**FIGURE 3:**
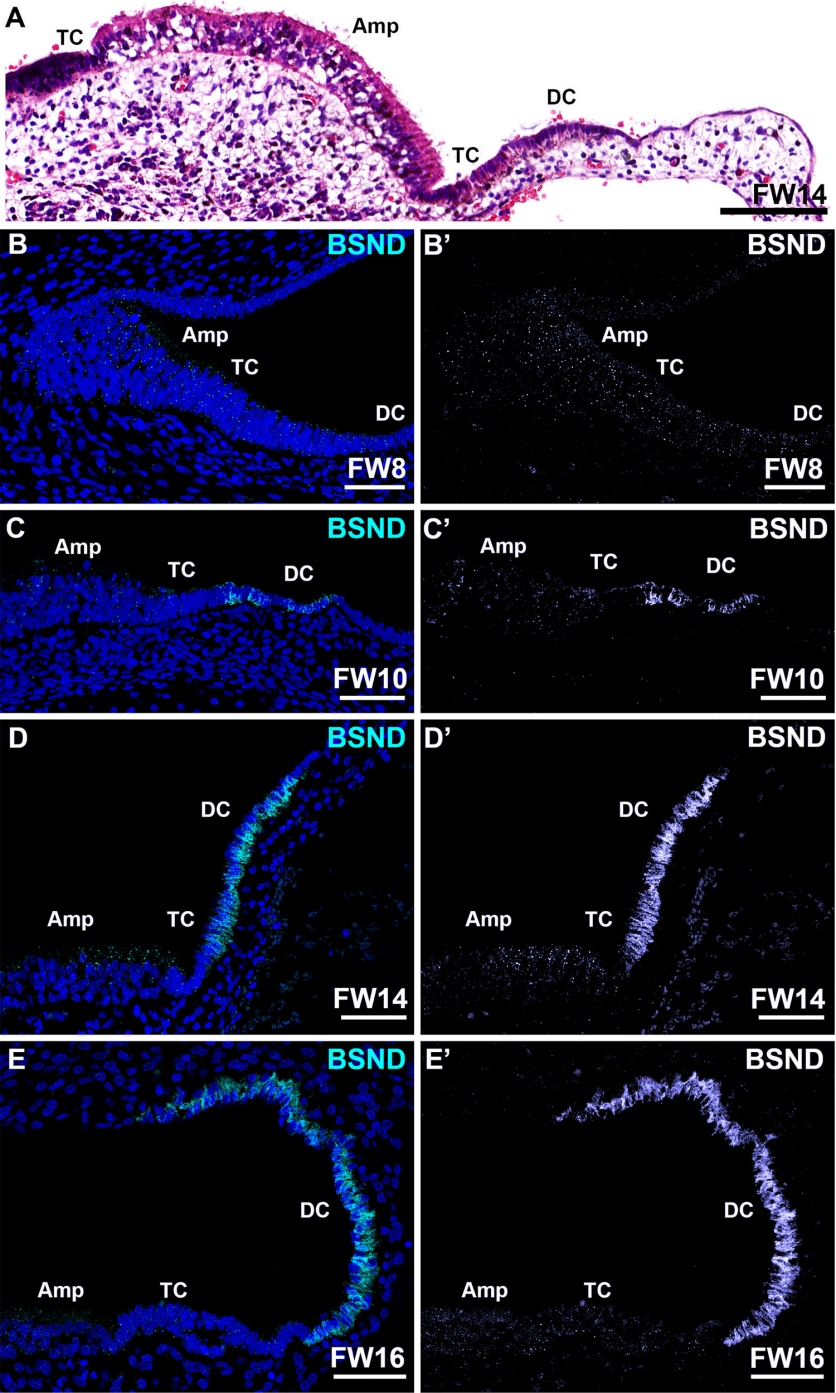
Expression of BSND in the developing ampulla. (A) H&E overview of the developing vestibular system at FW10. (B–B’) At FW8, punctuated expression of BSND can be observed in epithelial cells of the ampulla. (C–C’) At FW10, the sensory domain continues to express BSND, whereas some transitional cells and the dark cells express immunostaining at their basolateral membranes. (D–D’) At FW14, strong immunostaining is obvious along the basolateral membranes of both transitional cells and dark cells. The sensory domain continues to show punctuated expression of BSND. (E–E’) At FW16, only dark cells show immunostaining at their basolateral membranes. The sensory domain expresses minor punctuated immunostaining for BSND. Amp, sensory domain of the ampulla; DC, dark cells; TC, transitional cells. Cyan: BSND; blue: DAPI. Scale bars: 100 µm (A), 50 µm (B–E’).

At FW8, diffuse and punctuated immunostaining for BSND was observed in the prosensory domain of the ampulla and in the surrounding epithelia (Figure 3A–B’). The punctuated pattern was consistently visible in the ampullary sensory domain from FW8 up through FW16 (Figure 3B–E’). At FW10, expression was also seen at the basolateral membranes of both transitional cells and dark cells in the ampulla (Figure 3C–C’). At FW14, the transitional cells and dark cells strongly immunostained for BSND at their basolateral membranes (Figure 3A,D–D’), whereas at FW16 the expression in the transitional cells had diminished (Figure 3E–E’).

### 3.3 Distribution of Gap Junction Proteins GJA1, GJB2, and GJB6 in the Developing Vestibular Organs

We studied the temporal expression of several gap junction proteins on fetal inner ears from FW8‐17. Of these proteins, only GJB2 (connexin 26) has been shown to be present in the human vestibular system, but GJB6 (connexin 30) and GJA1 (connexin 43) have not been investigated.

**FIGURE 4:**
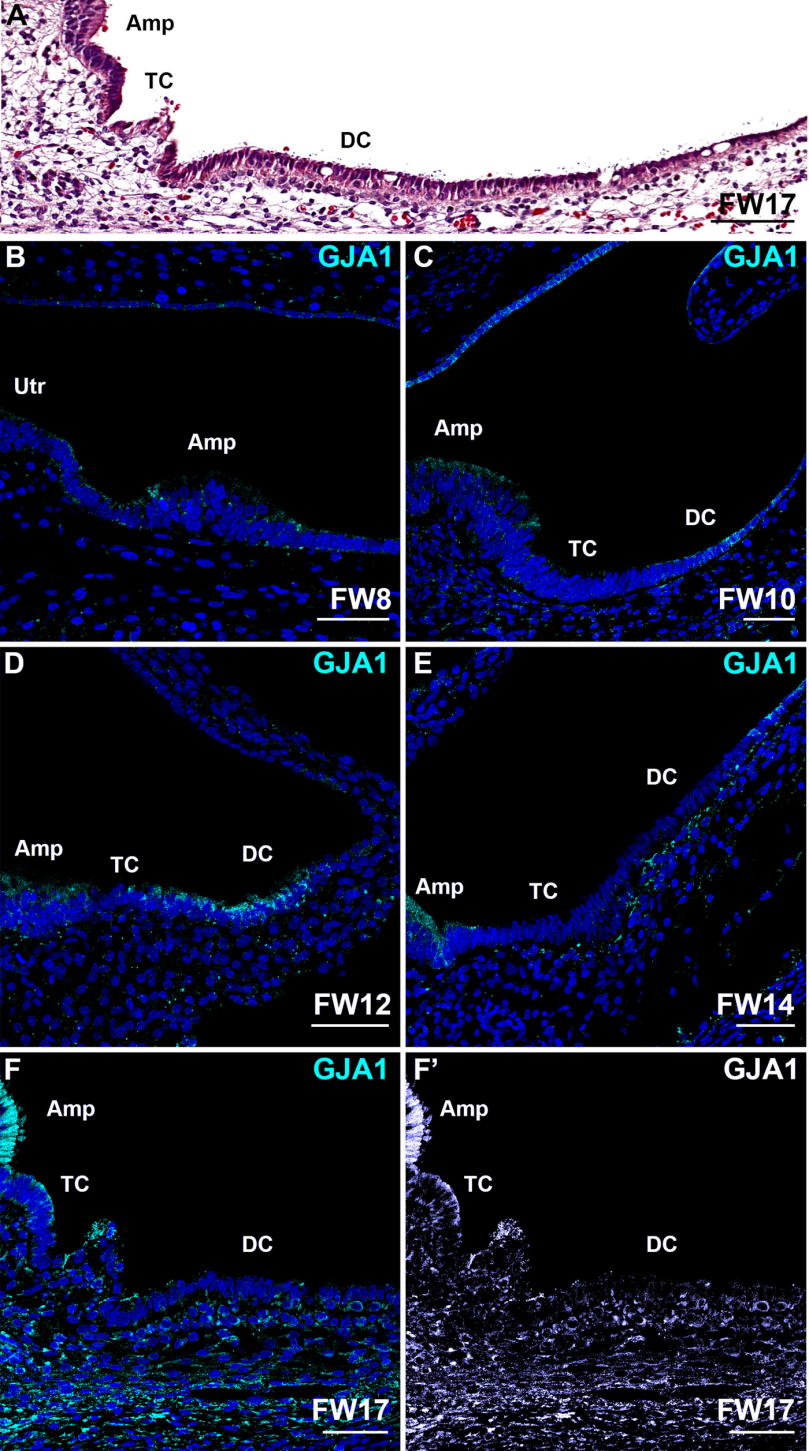
Expression of GJA1 is seen in a variety of vestibular cells. (A) H&E overview of the developing ampulla at FW17. (B) At FW8, low expression levels are seen at the edges of the prosensory domains of the ampulla and utricle. (C) At FW10, GJA1 is expressed by the dark cells. The sensory domain shows faint immunostaining. (D) At FW12, both the sensory domain and the dark cells show immunostaining at their apical membranes. Punctuated immunostaining is visible in the subepithelial mesenchyme. (E) At FW14, the dark cells show faint expression, whereas expression in subepithelial melanocytes and the sensory domain increases. (F–F’) At FW17, strong expression of GJA1 is seen in the sensory domain, the subepithelial mesenchyme and in the subepithelial melanocytes underneath the dark cell epithelium. Amp, sensory domain of the ampulla; DC, dark cells; TC, transitional cells Utr, sensory domain of the utricle. Cyan: GJA1; blue: DAPI. Scale bars: 100 µm (A), 50 µm (B–F’).

Expression of GJA1 was seen in a variety of vestibular cells (Figure 4). At FW8, few epithelial cells surrounding the prosensory domain of the ampulla and utricle and in the ampullar roof immunostained for GJA1, as well as the subepithelial mesenchyme (Figure 4A,B). At FW10, expression of GJA1 was seen in the developing dark cells surrounding the ampulla, in the sensory domain of the ampulla, and in some non‐specific epithelial cells. Faint immunostaining was seen in the transitional zone and some periotic mesenchyme (Figure 4C). At FW12, expression of GJA1 was more defined in the dark cell area, as compared to earlier weeks. Punctuated expression was seen in the subepithelial mesenchyme (Figure 4D). At FW14, expression was first seen in melanocytes underneath the dark cell epithelium (Figure 4E). At FW17, strong immunostaining was obvious in the sensory domain, the subepithelial melanocytes and mesenchyme, and the transitional cells (Figure 4A,F–F’).

**FIGURE 5:**
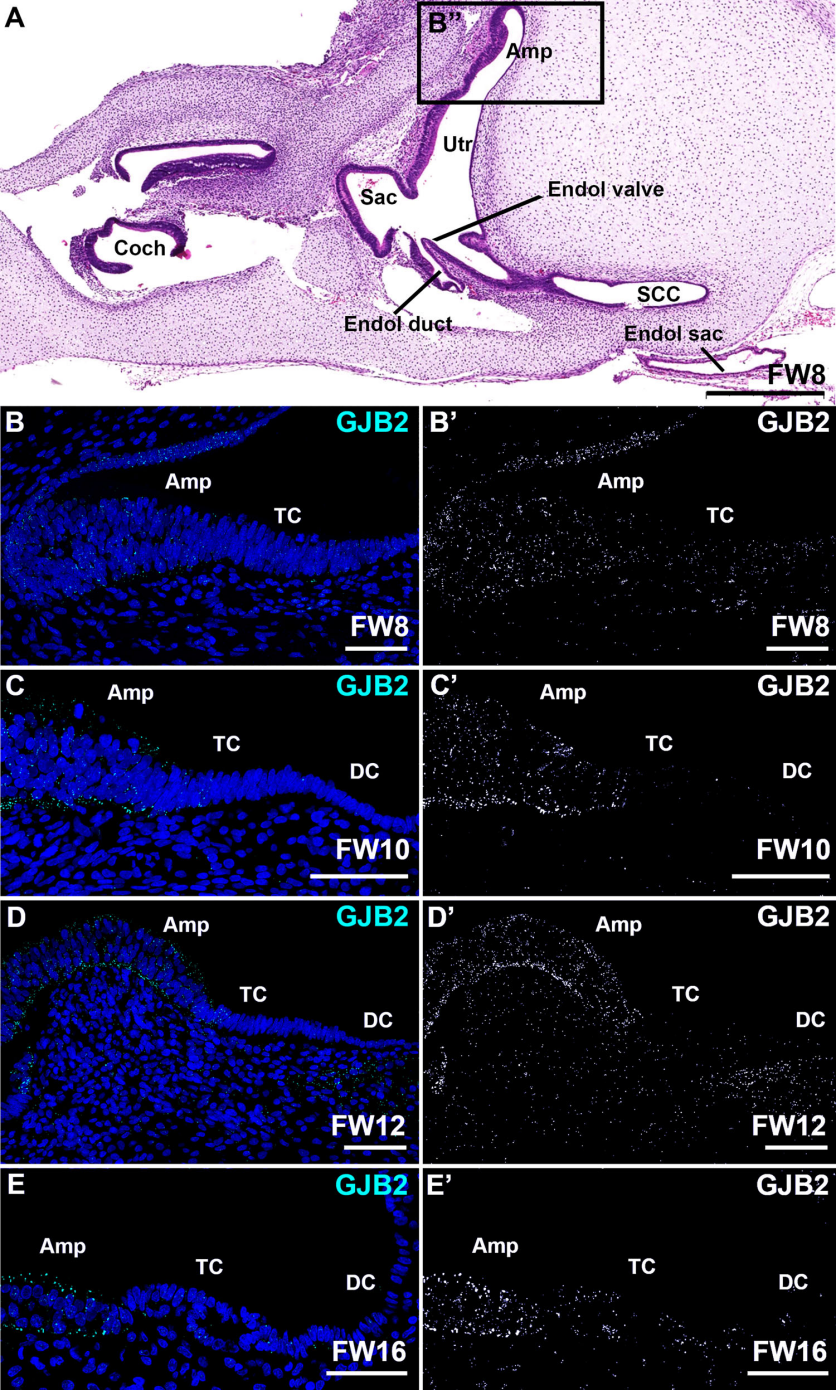
Expression of GJB2 is mostly restricted to the sensory domain of the ampulla and only diffusely present in the subepithelial mesenchyme underneath the transitional cells and the dark cells. (A) H&E overview of the developing inner ear at FW8. (B–B’) At FW8, only limited punctuated expression can be observed in the epithelial cells (C–C’). At FW10, immunostaining is visible in the sensory domain, whereas it is absent in the transitional cells and dark cells. (D–D’) At FW12, expression of GJB2 is seen in the sensory domain and the subepithelial mesenchyme. (E–E’) At FW14 (data not shown) and FW16, expression of GJB2 remains unchanged as compared to FW12. Amp, sensory domain of the ampulla; Coch, cochlea; DC, dark cells; Endol, endolymphatic (duct/sac/valve); Sac, sensory domain of the saccule; SCC, semicircular canal; TC, transitional cells; Utr, sensory domain of the utricle. Cyan: GJB2; blue: DAPI. Scale bars: 500 µm (A), 50 µm (B–E’).

**FIGURE 6:**
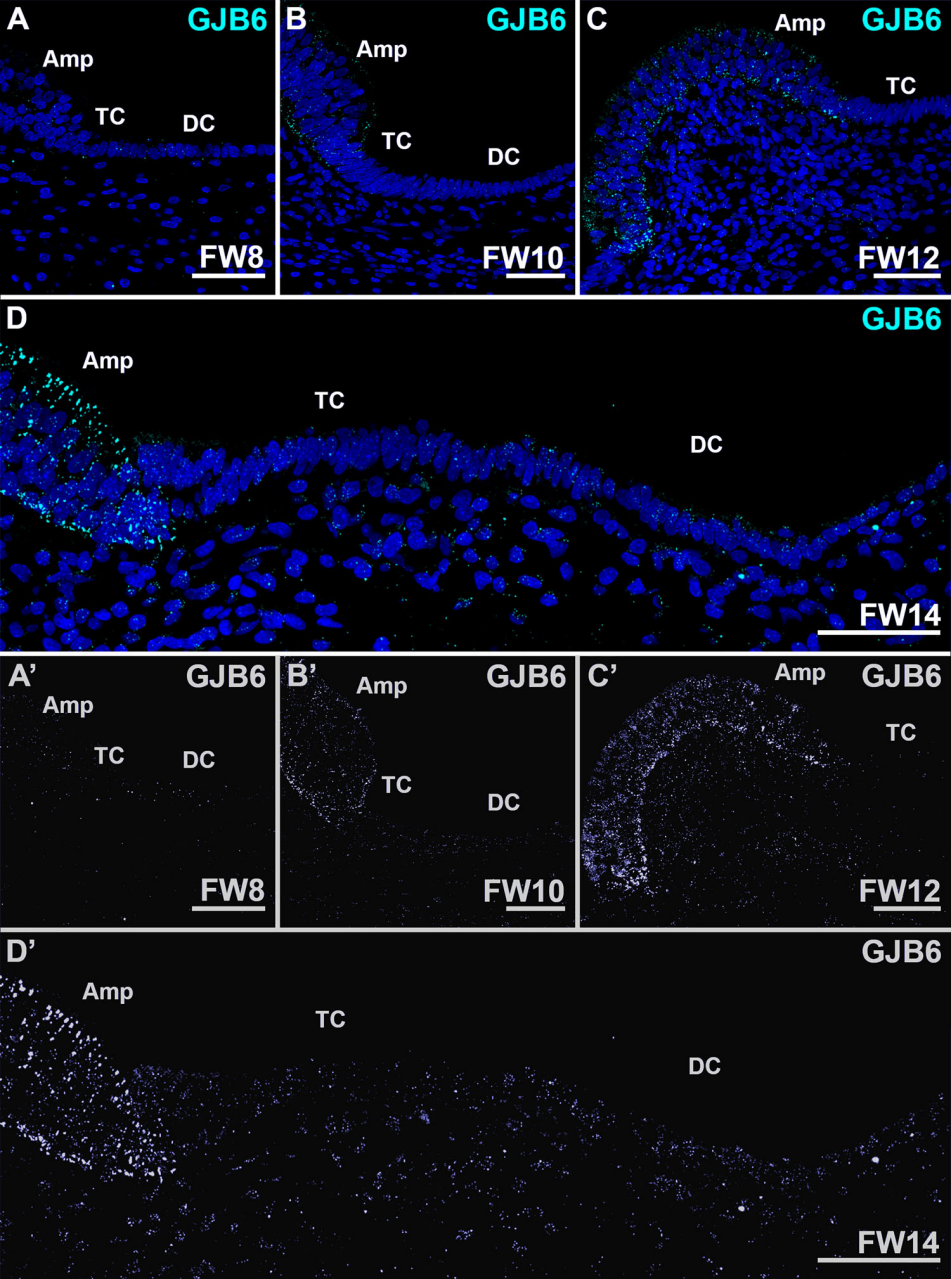
Expression of GJB6 is mostly restricted to the sensory domain of the ampulla and diffusely distributed to the subepithelial mesenchyme underneath the transitional cells and dark cells. (A, A’) At FW8, only few punctae are visible in the ampullar epithelial cells. (B, B’) At FW10, more immunostaining is visible in the sensory domain, whereas expression is absent in the developing transitional cells and the dark cells. (C, C’) At FW12, expression of GJB6 is seen in the sensory domain and subepithelial mesenchyme. (D, D’) The expression pattern of GJB2 remains unchanged as compared to FW12. Amp, sensory domain of the ampulla; DC, dark cells; TC, transitional cells. Cyan: GJB2; blue: DAPI. Scale bars: 50 µm.

At all developmental stages, GJB2 and GJB6 showed identical expression patterns (Figures 5 and 6). At FW8, immunostaining for GJB2 and GJB6 showed a diffuse and punctuated pattern in the sensory and extrasensory epithelia of the ampulla (Figure 5A–B’ and Figure 6A–A’). At FW10, immunostaining increased in the basolateral and apical membranes of the sensory domain (Figure 5C–C’, Figure 6B–B’). From FW12‐16, expression of GJB2 and GJB6 (FW16 data not shown) remained consistently visible in the sensory domain of the ampulla and the subepithelial mesenchyme (Figure 5D–E’; Figure 6C–D’; 6C’–D’). Expression patterns of both connexins were similar at FW16 and FW17 in the developing utricle (data not shown).

### 3.4 Transitional Cells and Dark Cells Express K^+^ Channel Protein KCNQ1 at Their Apical Membranes Around FW14

**FIGURE 7:**
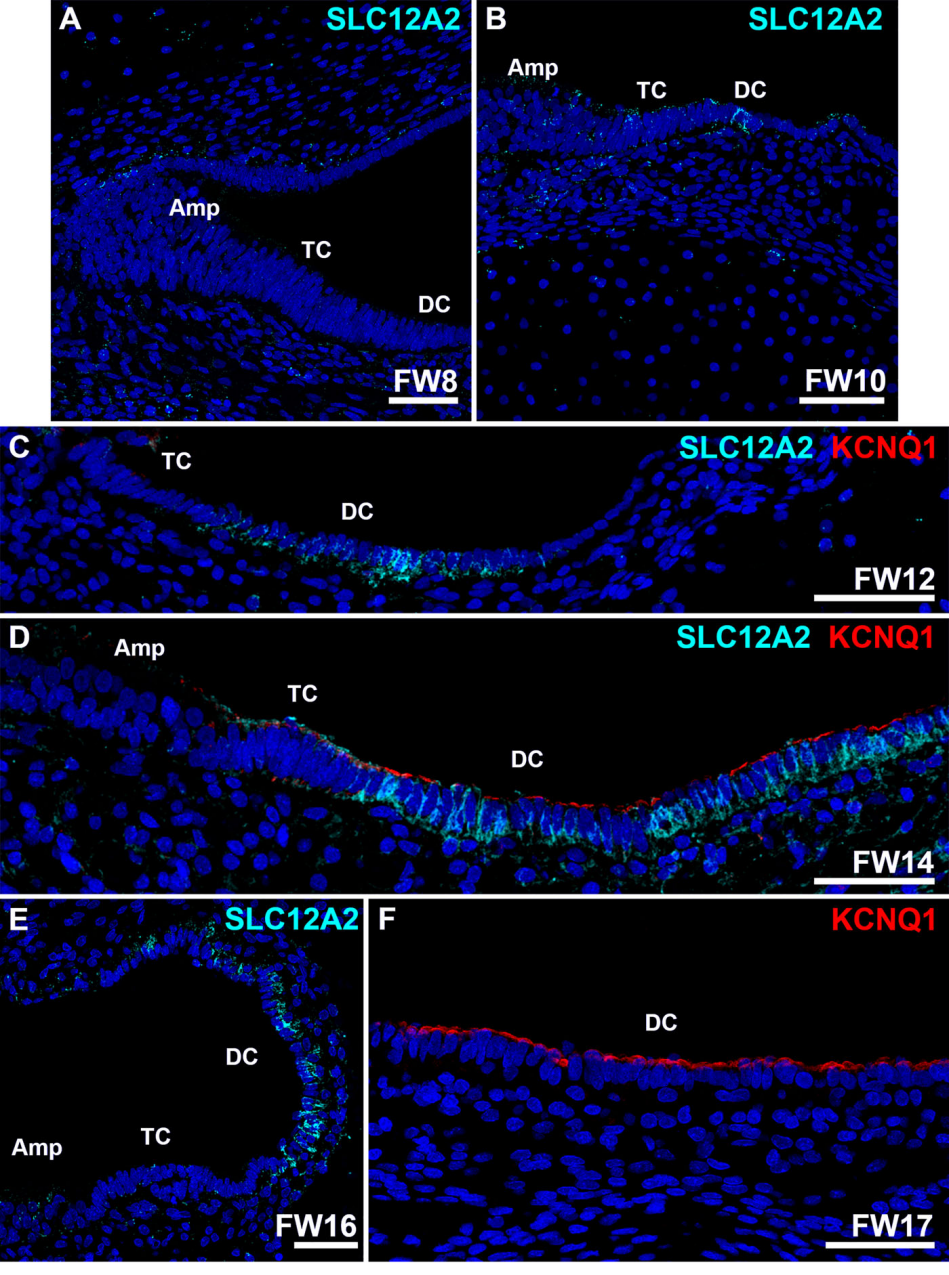
Ampullar dark cells start to express SLC12A2 around FW10 and KCNQ1 around FW14. (A) At FW8, expression of SLC12A2 is obvious as diffuse punctae in the subepithelial mesenchyme. (B) At FW10, some epithelial cells in the dark cell area start to express SLC12A2 at their basolateral membranes. (C). At FW12, expression in membranes of dark cells is increased as compared to FW10. KCNQ1 is not seen at this stage. (D) At FW14, strong immunostaining of SLC12A2 is seen in the basolateral membranes of the ampullar dark cells. At this stage, KCNQ1 is first observed in the apical membranes of the dark cell epithelium. (E) At FW16, expression of SLC12A2 is unchanged compared to FW14. No immunostaining of sensory epithelia is seen. (F) Apical membranes of ampullar dark cells immunostain intensely for KCNQ1 at FW17. Amp, ampulla; DC, dark cells; TC, transitional cells. Cyan: SLC12A2; red: KCNQ1; blue: DAPI. Scale bars: 50 µm.

From FW8‐12, expression of KCNQ1 was not seen in the developing vestibular organs (Figures 7A–C). At FW14, both dark cells and transitional cells of the ampulla showed immunostaining of KCNQ1 at their apical membranes (Figure 7D). At FW16, expression was confined to the apical membranes of the dark cells in the ampulla (Figure 7F) and utricle (data not shown). No expression of KCNQ1 was seen in other epithelial cells (data not shown).

### 3.5 Na^+^/K^+^/2Cl^−^ Cotransporter Protein SLC12A2 Is Expressed by Dark Cells Around FW10

From FW8‐10, diffuse and punctuated immunostaining for SLC12A2 (NKCC1) was seen in the subepithelial mesenchyme of the ampulla (Figure 7A,B). Immunostaining at the basolateral membranes of the dark cells surrounding the ampulla was first seen around W10 (Figure 7B). From FW12‐16, the expression pattern of the basolateral membranes of the ampullar dark cells became more evident (Figure 7C–E). No immunostaining was seen in the sensory domain of the ampulla (Figure 7D,E).

### 3.6 Temporal Expression of Pendrin Protein SLC26A4 in the Developing Vestibular Organs

**FIGURE 8:**
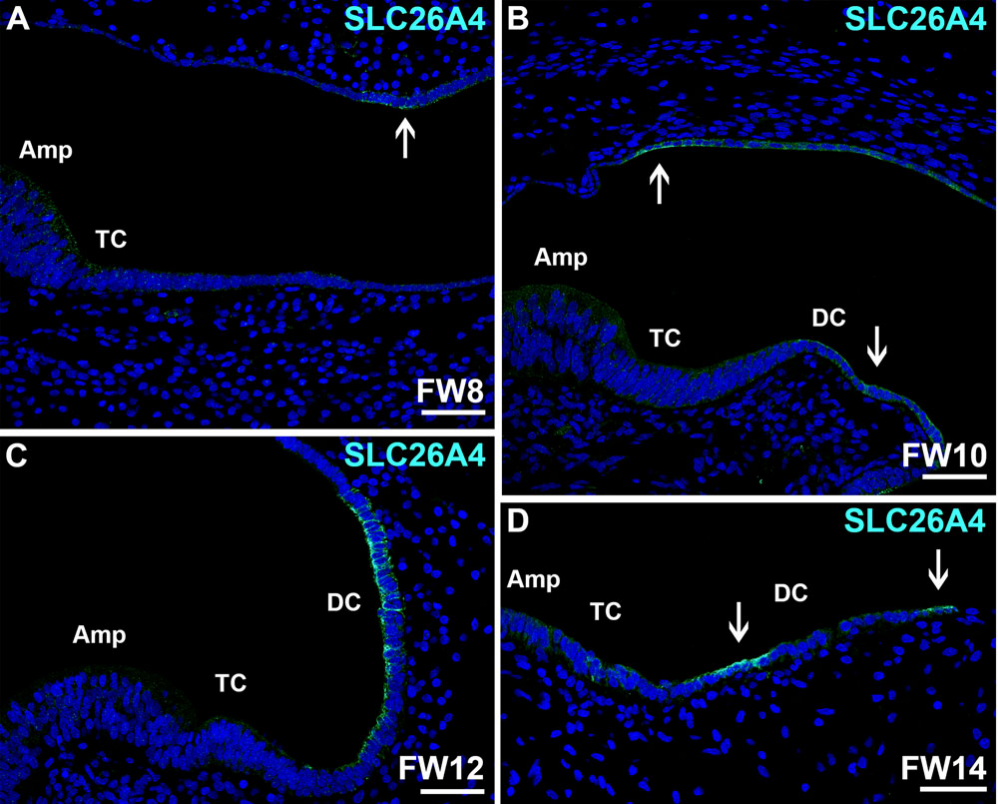
Immunostaining of SLC26A4 follows a dynamic pattern. (A) At FW8, apical membranes of epithelial cells in the ampullar roof immunostain for SLC26A4 (arrow). (B) At FW10, expression becomes more evident in both apical and basolateral membranes of utricular and ampullar dark cells. (C) At FW12, apical and lateral membranes of the dark cells immunostain for SLC26A4. (D) At FW14, dark cells (arrows) and transitional cells show apical expression. Amp, ampulla; DC, dark cells; TC, transitional cells. Cyan: SLC26A4; blue: DAPI. Scale bars: 50 µm.

At FW8, some epithelial cells in the ampullar roof expressed SLC26A4 (pendrin) at their basolateral and apical membranes (Figure 8A). At FW10, expression was more evident and was observed at the apical and basolateral membranes of dark cells located in the area between the utricle and ampulla as well as in the ampullar roof (Figure 8B). At FW12, immunostaining was intense and restricted to the apical and lateral membranes of the dark cell epithelia, and faint expression was observed at the apical membranes of the transitional cells (Figure 8C). At FW14, immunostaining for SLC26A4 was seen at the apical membranes of dark cells and to a lesser degree in the apical membranes of transitional epithelia (Figure 8D).

The spatio‐temporal expression patterns of the investigated proteins are summarized in Table 2.

**TABLE 2:** Spatio‐temporal expression patterns of investigated proteins in the developing human vestibular organs.

ATP1A1	FW10‐14: sensory domain, transitional cells, dark cells, subepithelial mesenchyme FW16: sensory domain, dark cells, subepithelial mesenchyme
ATP1B2	FW10: dark cells, transitional cells FW14‐16: dark cells
BSND	FW8: prosensory domain, extrasensory epithelia FW10‐14: transitional cells, dark cells, sensory domain FW16: dark cells, faint expression in sensory domain and transitional cells
GJA1	FW8: extrasensory domain, mesenchyme FW10‐12: sensory domain, mesenchyme, transitional cells, dark cells FW14: as above, including melanocytes FW17: sensory domain, transitional cells, mesenchyme, melanocytes
GJB2	FW8: prosensory domain, extrasensory epithelia FW10: sensory domain FW12‐16: sensory domain, mesenchyme
GJB6	FW8: prosensory domain, extrasensory epithelia FW10: sensory domain FW12‐16: sensory domain, mesenchyme
KCNQ1	FW8‐12: no expression FW14: dark cells, transitional cells FW16: dark cells
SLC12A2	FW8‐10: mesenchyme FW10‐16: dark cells, mesenchyme, transitional cells
SLC26A4	FW8‐14 dark cells, transitional cells

## 4 Discussion

### 4.1 Expression of Gap Junctions in the Developing Vestibular System

Mutations in the genes *GJA1*, *GJB2*, and *GJB6* that code for gap junction proteins Cx43, Cx26, and Cx30, respectively, are related to hearing loss (Abitbol et al. 2018; X. Z. Liu et al. 2001; Shearer et al. 1993) but so far only mutations in *GJB2* have been associated with vestibular symptoms in humans (Dodson et al. 2011; Kasai et al. 2010; Tsukada et al. 2015). While the expression patterns of these proteins have been studied in the human cochlea (W. Liu et al. 2009; Locher et al. 2015), they remain unexplored in human vestibular organs. In this study, we have investigated the expression of GJA1, GJB2, and GJB6 in the developing human vestibular organs, from FW8‐17. Given that cochlear expression patterns of these genes generally do not significantly differ between rodent species and humans, we anticipated similar findings in human vestibular organs as previously described in mice, gerbils, and guinea pigs (Forge et al. 2003). Indeed, our results showed that GJB2 and GJB6 exhibited identical expression patterns in the developing human vestibular organs, consistent with earlier findings demonstrating their co‐localization and heteromeric and heterotypic combinations (Lautermann et al. 1998).

Furthermore, vestibular melanocytes, but not dark cells, in the human adult inner ear have been shown to express GJB2 (Masuda et al. 2001; Wang et al. 2024). We did not find any expression of GJB2 in vestibular melanocytes until FW17. This suggests that its expression occurs at later developmental stages. In addition, developing vestibular epithelia, both sensory and extrasensory, expressed GJA1, contrasting with the expression in developing cochlear epithelia (Locher et al. 2015). At FW17, melanocytes expressed GJA1, unlike the intermediate cells (i.e., melanocytes of the stria vascularis) in the cochlea at the same developmental stage (Locher et al. 2015). GJA1 has been shown to be functionally significant in hearing (X. Z. Liu et al. 2001), suggesting that its cochlear expression occurs at later stages of development.

### 4.2 Dark Cells: Developmental Expression of Ion Transporters and Channels

From FW10 onwards, ATP1A1 and ATP1B2 co‐localized in the basolateral membranes of dark cells. In addition, BSND and SLC12A2 were first observed in the basolateral membranes of transitional and dark cells at FW10, with increasing intensity over time. Our observations are consistent with previous studies conducted by our group, which demonstrate that the vestibular system exhibits a developmental progression that precedes that of the cochlea by 2–3 weeks (Locher et al. 2015, 2013; van Beelen et al. 2020; van der Valk et al. 2023).

Interestingly, KCNQ1 expression in the apical membranes of dark cells was not detected until FW14, corresponding to its temporal expression pattern in cochlear marginal cells (melanocytes), which begins at FW16 (Locher et al. 2015). In addition, SLC26A4 expression was detected in the apical membranes of dark cells as early as FW8, reaching a peak at FW12 before declining. In the developing mouse inner ear, pendrin plays a crucial role in maintaining a balanced endolymph pH and regulating fluid absorption and secretion (Wangemann 2011). In humans, mutations in *SLC26A4* cause hearing loss and vestibular dysfunction manifesting at an early age (Honda and Griffith 2022). The observed gradient of expression in the human vestibular system might suggest a critical window for SLC26A4.

### 4.3 Transitional Cells: Contribution to Early Endolymph Homeostasis and K^+^ Regulation?

During early development, ion transporters and channels exhibit broader expression patterns, observed also in transitional cells before becoming restricted to dark cells. In mice, transitional cells contribute to early endolymph homeostasis when dark cells have not reached their mature phenotype yet (Bartolami et al. 2011). In this study, we found that ATP1A1, ATP1B2, BSDN, SLC12A2, and SLC26A4 were expressed by both transitional cells and dark cells during early development, similar to their developmental profile in mice. Previously, we have shown that early during human vestibular development, melanocytes not only associate with dark cell epithelia, but with transitional cells as well (van Beelen et al. 2020). Thus, it is possible that transitional cells play a role in early endolymph homeostasis in the developing human vestibular organs.

### 4.4 Limitations

The findings stated here are based on a limited number of specimen. Specifically, for later stages of development, few samples were successfully processed. During maturation of the inner ear, the cartilaginous capsule ossifies, which poses challenges during the collection process. Most samples from FW14 onwards are damaged during the vacuum aspiration. We compensated for the limited number of samples by using a robust internal control system and contextualizing the results against a background of extensive literature on ion channel development in rodent species and rabbits (Table 3). These findings support the conclusion that the most dynamic and critical spatiotemporal expression window for ion transport occurs in early stages between FW8‐14.

**TABLE 3:** Ion transporters and channels in rodents and rabbits.

	ATP1A1	ATP1B2	BSND	KCNQ1	SLC12A2	SLC26A4
Mouse	(Schulte and Steel 1994)	(Schulte and Steel 1994)	(Birkenhäger et al. 2001; Estévez et al. 2001)	(Neyroud et al. 1997; Lee et al. 2000; Casimiro et al. 2001; Nicolas et al. 2001)	(Delpire et al. 1999)	(Everett et al. 1999; Royaux et al. 2003; Yoshino et al. 2004)
Rat	(Yao et al. 1994)	(ten Cate et al. 1994; Fina and Ryan 1994; Peters et al. 2001)	(Qu et al. 2007)	(Liang et al. 2006)	(Goto et al. 1997; Akiyama et al. 2010)	
Gerbil	(McGuirt and Chulte 1994)	(Schulte and Adams 1989; McGuirt and Chulte 1994)	(Sage and Marcus 2001)		(Crouch et al. 1997; Sakaguchi et al. 1998)	
Guinea pig				(Liang et al. 2006)		
Rabbit					(Miuta et al. 1997)	

## 5 Conclusion

We described the developmental expression of several membrane pumps and ion channels essential in vestibular function. The observed dynamic profiles underscore the intricate process of ion homeostasis in the developing vestibular system and might suggest a critical window for each investigated protein.

## Author Contributions

Conceptualization, JG, PB, HL. Funding acquisition, JG, PB, HL. Investigation, EB, WV. Methodology, all authors. Writing – original draft, all authors. Writing – review and editing, EB, WV, PB, HL.

## Conflicts of Interest

The authors declare no conflicts of interest.

## Supporting information




**Supplementary Figure S1‐S2**: dneu70010‐sup‐0001‐SuppMat.docx

## Data Availability

All data generated or analyzed during this study are available from the corresponding author upon reasonable request.

## References

[dneu70010-bib-0001] Abitbol, J. M. , J. J. Kelly , K. J. Barr , B. L. Allman , and D. W. Laird . 2018. “Mice Harbouring an Oculodentodigital Dysplasia‐Linked Cx43 G60S Mutation Have Severe Hearing Loss.” Journal of Cell Science 131, no. 9: jcs214635. 10.1242/jcs.214635. 29618634

[dneu70010-bib-0002] Akiyama, K. , T. Miyashita , A. Matsubara , and N. Mori . 2010. “The Detailed Localization Pattern of Na^+^/K^+^/2Cl^−^ Cotransporter Type 2 and Its Related Ion Transport System in the Rat Endolymphatic Sac.” Journal of Histochemistry & Cytochemistry 58, no. 8: 759–763. 10.1369/jhc.2010.956045. 20458062 PMC2907281

[dneu70010-bib-0003] Bartolami, S. , S. Gaboyard , J. Quentin , et al. 2011. “Critical Roles of Transitional Cells and Na/K‐ATPase in the Formation of Vestibular Endolymph.” Journal of Neuroscience 31, no. 46: 16541–16549. 10.1523/JNEUROSCI.2430-11.2011. 22090480 PMC6633285

[dneu70010-bib-0004] Birkenhäger, R. , E. Otto , M. J. Schürmann , et al. 2001. “Mutation of BSND Causes Bartter Syndrome With Sensorineural Deafness and Kidney Failure.” Nature Genetics 29, no. 3: 310–314. 10.1038/ng752. 11687798

[dneu70010-bib-0005] Casimiro, M. C. , B. C. Knollmann , S. N. Ebert , et al. 2001. “Targeted Disruption of the Kcnq1 Gene Produces a Mouse Model of Jervell and Lange–Nielsen Syndrome.” Proceedings of the National Academy of Sciences of the United States of America 98, no. 5: 2526–2531. 10.1073/pnas.041398998. 11226272 PMC30171

[dneu70010-bib-0006] Ten Cate, W.‐J. F. , L. M. Curtis , and K. E. Rarey . 1994. “Na,K‐ATPase α and β Subunit Isoform Distribution in the Rat Cochlear and Vestibular Tissues.” Hearing Research 75, no. 1–2: 151–160. 10.1016/0378-5955(94)90066-3. 8071142

[dneu70010-bib-0007] Crouch, J. J. , N. Sakaguchi , C. Lytle , and B. A. Schulte . 1997. “Immunohistochemical Localization of the Na‐K‐Cl co‐Transporter (NKCC1) in the Gerbil Inner Ear.” Journal of Histochemistry & Cytochemistry 45, no. 6: 773–778. 10.1177/002215549704500601. 9199662

[dneu70010-bib-0008] Delpire, E. , J. Lu , R. England , C. Dull , and T. Thorne . 1999. “Deafness and Imbalance Associated With Inactivation of the Secretory Na‐K‐2Cl Co‐Transporter.” Nature Genetics 22, no. 2: 192–195. 10.1038/9713. 10369265

[dneu70010-bib-0009] Dodson, K. M. , S. H. Blanton , K. O. Welch , et al. 2011. “Vestibular Dysfunction in DFNB1 Deafness.” American Journal of Medical Genetics, Part A 155, no. 5: 993–1000. 10.1002/ajmg.a.33828. PMC3080433 21465647

[dneu70010-bib-0010] Estévez, R. , T. Boettger , V. Stein , et al. 2001. “Barttin Is a Cl^−^ Channel β‐subunit Crucial for Renal Cl^−^ Reabsorption and Inner Ear K^+^ Secretion.” Nature 414, no. 6863: 558–561. 10.1038/35107099. 11734858

[dneu70010-bib-0011] Everett, L. A. , H. Morsli , D. K. Wu , and E. D. Green . 1999. “Expression Pattern of the Mouse Ortholog of the Pendred's Syndrome Gene (Pds) Suggests a Key Role for Pendrin in the Inner Ear.” Proceedings of the National Academy of Sciences of the United States of America 96, no. 17: 9727–9732. 10.1073/pnas.96.17.9727. 10449762 PMC22278

[dneu70010-bib-0012] Fina, M. , and A. Ryan . 1994. “Expression of mRNAs Encoding α and β Subunit Isoforms of Na,K‐ATPase in the Vestibular Labyrinth and Endolymphatic Sac of the Rat.” Molecular and Cellular Neuroscience 5, no. 6: 604–613. 10.1006/mcne.1994.1074. 7704435

[dneu70010-bib-0013] Forge, A. , D. Becker , S. Casalotti , J. Edwards , N. Marziano , and G. Nevill . 2003. “Gap Junctions in the Inner Ear: Comparison of Distribution Patterns in Different Vertebrates and Assessment of Connexin Composition in Mammals.” Journal of Comparative Neurology 467, no. 2: 207–231. 10.1002/cne.10916. 14595769

[dneu70010-bib-0014] Goto, S. , T. Oshima , K. Ikeda , N. Ueda , and T. Takasaka . 1997. “Expression and Localization of the Na‐K‐2Cl Cotransporter in the Rat Cochlea.” Brain Research 765, no. 2: 324–326. 10.1016/S0006-8993(97)00679-3. 9313906

[dneu70010-bib-0015] Honda, K. , and A. J. Griffith . 2022. “Genetic Architecture and Phenotypic Landscape of SLC26A4‐Related Hearing Loss.” Human Genetics 141, no. 3–4: 455–464. 10.1007/s00439-021-02311-1. 34345941

[dneu70010-bib-0016] Kasai, M. , C. Hayashi , T. Iizuka , et al. 2010. “Vestibular Function of Patients With Profound Deafness Related to GJB2 Mutation.” Acta Oto‐Laryngologica 130, no. 9: 990–995. 10.3109/00016481003596508. 20377502

[dneu70010-bib-0017] Köppl, C. , V. Wilms , I. J. Russell , and H. G. Nothwang . 2018. “Evolution of Endolymph Secretion and Endolymphatic Potential Generation in the Vertebrate Inner Ear.” Brain, Behavior and Evolution 92, no. 1–2: 1–31. 10.1159/000494050. 30415265

[dneu70010-bib-0018] Lautermann, J. , W. J. F. ten Cate , P. Altenhoff , et al. 1998. “Expression of the Gap‐Junction Connexins 26 and 30 in the Rat Cochlea.” Cell and Tissue Research 294, no. 3: 415–420. 10.1007/s004410051192. 9799458

[dneu70010-bib-0019] Lee, M. P. , J. D. Ravenel , R. J. Hu , et al. 2000. “Targeted Disruption of the Kvlqt1 Gene Causes Deafness and Gastric Hyperplasia in Mice.” Journal of Clinical Investigation 106, no. 12: 1447–1455. 10.1172/JCI10897. 11120752 PMC387258

[dneu70010-bib-0020] Liang, G. H. , Z. Jin , M. Ulfendahl , and L. Järlebark . 2006. “Molecular Analyses of KCNQ1‐5 Potassium Channel mRNAs in Rat and Guinea Pig Inner Ears: Expression, Cloning, and Alternative Splicing.” Acta Oto‐Laryngologica 126, no. 4: 346–352. 10.1080/00016480500416777. 16608784

[dneu70010-bib-0021] Liu, W. , M. Boström , A. Kinnefors , and H. Rask‐Andersen . 2009. “Unique Expression of Connexins in the Human Cochlea.” Hearing Research 250, no. 1–2: 55–62. 10.1016/j.heares.2009.01.010. 19450429

[dneu70010-bib-0022] Liu, X. Z. , X. J. Xia , J. Adams , et al. 2001. “Mutations in GJA1 (connexin 43) Are Associated With Non‐Syndromic Autosomal Recessive Deafness.” Human Molecular Genetics 10, no. 25: 2945–2951. 10.1093/hmg/10.25.2945. 11741837

[dneu70010-bib-0023] Locher, H. , J. C. M. J. de Groot , L. van Iperen , M. A. Huisman , J. H. M. Frijns , and S. M. Chuva de Sousa Lopes . 2015. “Development of the Stria Vascularis and Potassium Regulation in the Human Fetal Cochlea: Insights Into Hereditary Sensorineural Hearing Loss.” Developmental Neurobiology 75, no. 11: 1219–1240. 10.1002/dneu.22279. 25663387 PMC5024031

[dneu70010-bib-0024] Locher, H. , J. H. Frijns , L. van Iperen , J. C. de Groot , M. A. Huisman , and S. M. Chuva de Sousa Lopes . 2013. “Neurosensory Development and Cell Fate Determination in the Human Cochlea.” Neural Development 8, no. 1: 20. 10.1186/1749-8104-8-20. 24131517 PMC3854452

[dneu70010-bib-0025] Masuda, M. , S. Usami , K. Yamazaki , et al. 2001. “Connexin 26 Distribution in Gap Junctions Between Melanocytes in the Human Vestibular Dark Cell Area.” Anatomical Record 262, no. 2: 137–146. 10.1002/1097-0185(20010201)262:2<137::AID-AR1018>3.0.CO;2-2. 11169908

[dneu70010-bib-0026] McGuirt, J. P. , and B. A. Chulte . 1994. “Distribution of Immunoreactive α‐ and β‐Subunit Isoforms of Na,K‐ATPase in the Gerbil Inner Ear.” Journal of Histochemistry and Cytochemistry 42, no. 7: 843–853. 10.1177/42.7.8014467. 8014467

[dneu70010-bib-0027] Mei, C. , H. Dong , E. Nisenbaum , et al. 2021. “Genetics and the Individualized Therapy of Vestibular Disorders.” Frontiers in Neurology 12: 633207. 10.3389/fneur.2021.633207. 33613440 PMC7892966

[dneu70010-bib-0028] Miuta, K. , M. Adachi , and K. H. Iwasa . 1997. “Ultrastructural Localization of the Na‐K‐Cl Cotransporter in the Lateral Wall of the Rabbit Cochlear Duct.” Hearing Research 106, no. 1–2: 154–162. 10.1016/S0378-5955(97)00010-5. 9112115

[dneu70010-bib-0029] Neyroud, N. , F. Tesson , I. Denjoy , et al. 1997. “A Novel Mutation in the Potassium Channel Gene KVLQT1 Causes the Jervell and Lange‐Nielsen Cardioauditory Syndrome.” Nature Genetics 15, no. 2: 186–189. 10.1038/ng0297-186. 9020846

[dneu70010-bib-0030] Nicolas, M.‐T. , D. Demêmes , A. Martin , S. Kupershmidt , and J. Barhanin . 2001. “KCNQ1/KCNE1 Potassium Channels in Mammalian Vestibular Dark Cells.” Hearing Research 153, no. 1–2: 132–145. 10.1016/S0378-5955(00)00268-9. 11223304

[dneu70010-bib-0031] Peters, T. A. , W. Kuijpers , and J. H. A. J. Curfs . 2001. “Occurrence of NaK‐ATPase Isoforms During Rat Inner Ear Development and Functional Implications.” European Archives of Oto‐Rhino‐Laryngology 258, no. 2: 67–73. 10.1007/s004050000304. 11307608

[dneu70010-bib-0032] Qu, C. , F. Liang , N. M. Smythe , and B. A. Schulte . 2007. “Identification of ClC‐2 and CIC‐K2 Chloride Channels in Cultured Rat Type IV Spiral Ligament Fibrocytes.” Journal of the Association for Research in Otolaryngology: JARO 8, no. 2: 205–219. 10.1007/s10162-007-0072-0. 17334850 PMC2538358

[dneu70010-bib-0033] Royaux, I. E. , I. A. Belyantseva , T. Wu , et al. 2003. “Localization and Functional Studies of Pendrin in the Mouse Inner Ear Provide Insight About the Etiology of Deafness in Pendred Syndrome.” JARO—Journal of the Association for Research in Otolaryngology 4, no. 3: 394–404. 10.1007/s10162-002-3052-4. PMC3202734 14690057

[dneu70010-bib-0034] Sage, C. L. , and D. C. Marcus . 2001. “Immunolocalization of ClC‐K Chloride Channel in Strial Marginal Cells and Vestibular Dark Cells.” Hearing Research 160, no. 1–2: 1–9. 10.1016/S0378-5955(01)00308-2. 11591484

[dneu70010-bib-0035] Sakaguchi, N. , J. J. Crouch , C. Lytle , and B. A. Schulte . 1998. “Na‐K‐Cl Cotransporter Expression in the Developing and Senescent Gerbil Cochlea.” Hearing Research 118, no. 1–2: 114–122. 10.1016/S0378-5955(98)00022-7. 9606066

[dneu70010-bib-0036] Schulte, B. A. , and J. C. Adams . 1989. “Distribution of Immunoreactive Na+,K+‐ATPase in Gerbil Cochlea.” Journal of Histochemistry & Cytochemistry 37, no. 2: 127–134. 10.1177/37.2.2536055. 2536055

[dneu70010-bib-0037] Schulte, B. A. , and K. P. Steel . 1994. “Expression of α and β Subunit Isoforms of Na,K‐ATPase in the Mouse Inner Ear and Changes With Mutations at the Wv or Sld Loci.” Hearing Research 78, no. 1: 65–76. 10.1016/0378-5955(94)90045-0. 7961179

[dneu70010-bib-0038] Shearer, A. E. , M. S. Hildebrand , and R. J. Smith . 1993. “Hereditary Hearing Loss and Deafness Overview.” In GeneReviews®, edited by M. P. Adam , S. Bick , G. M. Mirzaa , R. A. Pagon , S. E. Wallace , and A. Amemiya . University of Washington. http://www.ncbi.nlm.nih.gov/pubmed/20301607.

[dneu70010-bib-0040] Tsukada, K. , H. Fukuoka , and S.‐I. Usami . 2015. “Vestibular Functions of Hereditary Hearing Loss Patients With GJB2 Mutations.” Audiology & Neuro‐Otology 20, no. 3: 147–152. 10.1159/000368292. 25824904

[dneu70010-bib-0041] van Beelen, E. S. A. , W. H. van der Valk , J. C. M. J. de Groot , E. F. Hensen , H. Locher , and P. P. G. van Benthem . 2020. “Migration and Fate of Vestibular Melanocytes During Development of the Human Inner Ear.” Developmental Neurobiology 80, no. 11–12: 411–432. 10.1002/dneu.22786. 33075185 PMC7894185

[dneu70010-bib-0042] van der Valk, W. H. , E. S. A. van Beelen , M. R. Steinhart , et al. 2023. “A Single‐Cell Level Comparison of Human Inner Ear Organoids With the Human Cochlea and Vestibular Organs.” Cell Reports 42, no. 6: 112623. 10.1016/j.celrep.2023.112623. 37289589 PMC10592453

[dneu70010-bib-0043] Wang, T. , A. H. Ling , S. E. Billings , et al. 2024. “Single‐Cell Transcriptomic Atlas Reveals Increased Regeneration in Diseased Human Inner Ear Balance Organs.” Nature Communications 15, no. 1: 4833. 10.1038/s41467-024-48491-y. PMC11156867 38844821

[dneu70010-bib-0044] Wangemann, P. 2002. “K(+) Cycling and Its Regulation in the Cochlea and the Vestibular Labyrinth.” Audiology & Neuro‐Otology 7, no. 4: 199–205. 10.1159/000063736. 12097719

[dneu70010-bib-0045] Wangemann, P. 2011. “The Role of Pendrin in the Development of the Murine Inner Ear.” Cellular Physiology and Biochemistry: International Journal of Experimental Cellular Physiology, Biochemistry, and Pharmacology 28, no. 3: 527–534. 10.1159/000335113. 22116367 PMC7077107

[dneu70010-bib-0046] Wangemann, P. , and D. C. Marcus . 2017. “Ion and Fluid Homeostasis in the Cochlea.” In Understanding the Cochlea, edited by G. A. Manley , A. W. Gummer , A. N. Popper , and R. R. Fay , 253–286. Springer. 10.1007/978-3-319-52073-5_9.

[dneu70010-bib-0047] Yao, X. , W. J. F. ten Cate , L. M. Curtis , and K. E. Rarey . 1994. “Expression of Na+,K+‐ATPase α1 Subunit mRNA in the Developing Rat Cochlea.” Hearing Research 80, no. 1: 31–37. 10.1016/0378-5955(94)90006-X. 7852201

[dneu70010-bib-0048] Yoshino, T. , E. Sato , T. Nakashima , et al. 2004. “The Immunohistochemical Analysis of Pendrin in the Mouse Inner Ear.” Hearing Research 195, no. 1–2: 9–16. 10.1016/j.heares.2004.05.005. 15350275

