# Supplementary material for: Developmental Expression of Membrane Pumps and Ion Channels in Human Vestibular Endolymph Homeostasis
Source: Dev Neurobiol. 2026 Jan 25;86(1):e70010. doi: 10.1002/dneu.70010 (PMC12832121; doi:10.1002/dneu.70010)

**Supplementary Figure 1.** Expression of ATP1A1 and ATP1B2 in the developing utricle and ampulla (high contrast image)

Panels A and B correspond to panel B of Figure 2. Panels C and D correspond to panel C of Figure 2. Panels E and F correspond to panel D of Figure 2. Panels G and H correspond to panel E of Figure 2. Amp, ampulla; DC, dark cells; TC, transitional cells; Utr, utricle. Greyscale: ATP1A1/ATP1B2. Scale bars: 50 µm.


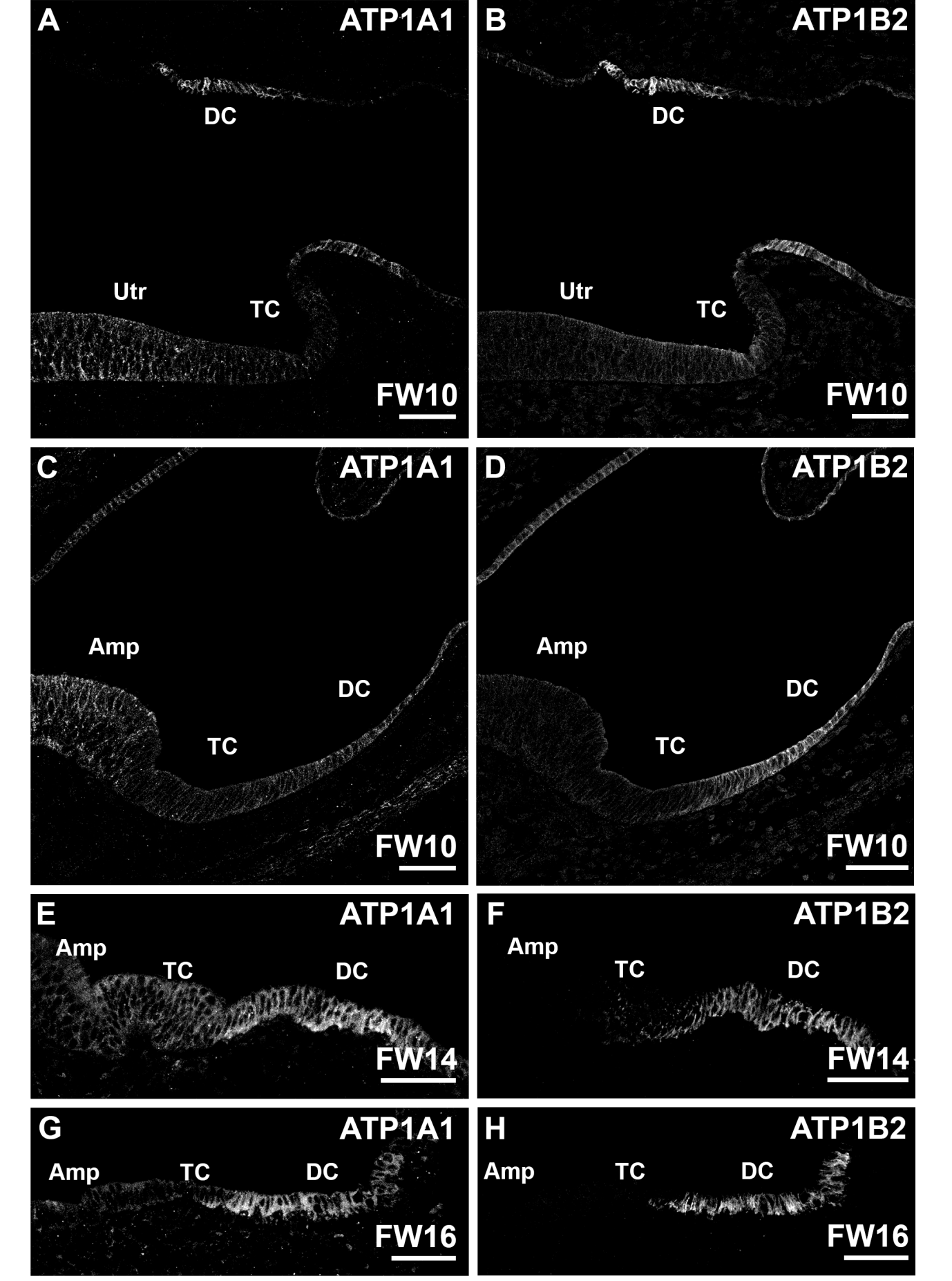


**Supplementary Figure 2.** Expression of SLC26A4 follows a dynamic pattern (high contrast image)

Panels A-D correspond to panels A-D of Figure 10. Amp, ampulla; DC, dark cells; TC, transitional cells. Greyscale: SLC26A4. Scale bars: 50 µm.


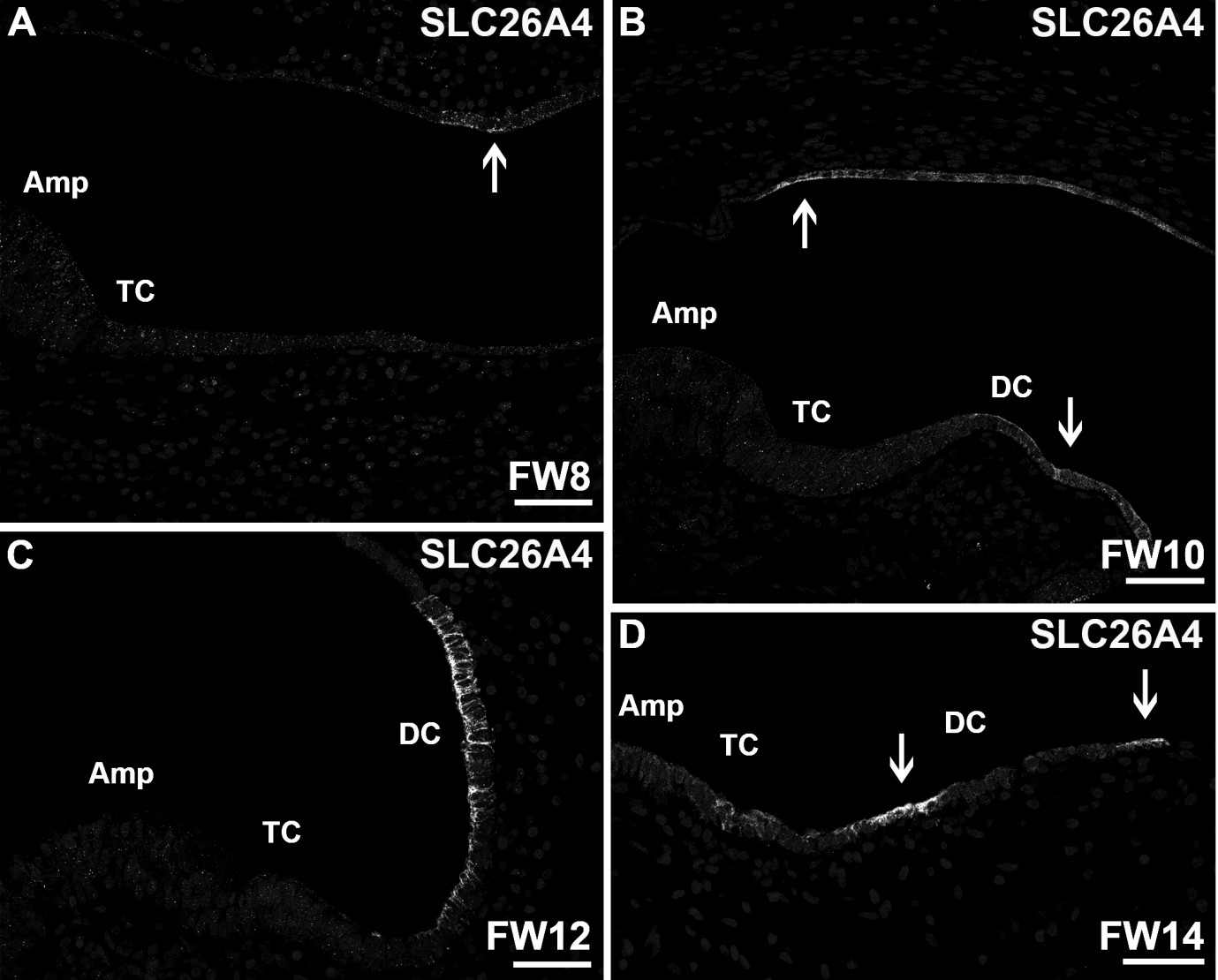

Supplement: Supplementary file 1 — Supplementary Figure S1‐S2: dneu70010‐sup‐0001‐SuppMat.docx [file DNEU-86-0-s001.docx]
